# Isoprene Production by *Sphagnum* Moss Is Balanced by Microbial Uptake, as Revealed by Selective Inhibitors

**DOI:** 10.1111/1462-2920.70114

**Published:** 2025-06-05

**Authors:** Andrew T. Crombie, Chloe L. Wright, Ornella Carrión, Laura E. Lehtovirta‐Morley, J. Colin Murrell

**Affiliations:** ^1^ School of Biological Sciences University of East Anglia Norwich UK; ^2^ School of Environmental Sciences University of East Anglia Norwich UK

**Keywords:** biogeochemical cycles, isoprene, methanotrophs, monooxygenase, peatlands, *Sphagnum*, volatiles

## Abstract

Northern peatlands, ecosystems which store enormous amounts of carbon, and yet are major sources of methane and plant‐derived volatiles including isoprene, are predicted to be greatly affected by climate change. Isoprene, the major volatile secondary metabolite released by plants, can support the carbon and energy needs of a variety of bacteria. Here we show that *Sphagnum* moss from an acidic bog harboured highly active isoprene degraders which consumed the vast majority of the plant‐produced isoprene, preventing its release to the atmosphere. We quantified the potential for microbial isoprene uptake in the moss and, using alkyne inhibitors specific to either isoprene monooxygenase of *bona fide* isoprene degraders, or to the enzymes of other microbes capable of its fortuitous co‐oxidation, we show that methane utilizers, for example, did not oxidise significant isoprene in incubations. Our technique enabled the separate quantification of plant isoprene production and microbial uptake, revealing that although atmospheric isoprene concentrations are typically low, the microbes contained in, or in close association with the moss were capable of isoprene uptake at the plant‐generated isoprene concentration. Analysis of the bacterial community suggested that the isoprene degraders in this environment belonged to novel groups distinct from extant strains with this capability.

## Graphical abstract

*Sphagnum* moss, typical of northern peatlands, produces a range of volatile organic compounds, principally the climate‐active compound, isoprene, which serves as a carbon source for specialist isoprene‐consuming microbes. Using an inhibitor of microbial isoprene uptake, we quantify gross isoprene production by the moss and show that the vast majority of moss‐produced isoprene is consumed by microbes, preventing its release to the atmosphere.

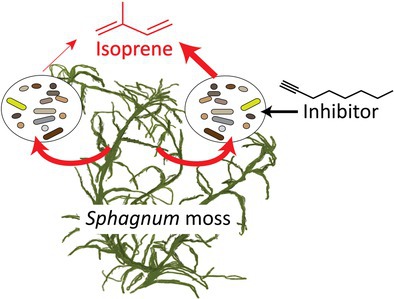

## 1 Introduction

Peatlands, wetland areas where the soil consists of partially decayed plant material, occur in northern, temperate and tropical regions and comprise approximately 3% of Earth's total land area. The majority of peatlands (about 80%) are in northern temperate, boreal and subarctic regions and despite their relatively modest global area, they store enormous amounts of carbon, formed mainly from decaying mosses and other peatland plants, equivalent to half the atmospheric carbon pool or 25%–50% of global soil carbon (Tarnocai et al. 2009). *Sphagnum* moss, described as the ‘builder of boreal peatlands’ by Rydin and Jeglum (2013) was speculated to contribute more to carbon stores globally than any other plant genus (Clymo and Hayward 1982), and is key to these ecosystems. However, wetlands, including peatlands, are the largest natural source of the potent greenhouse gas methane, and account for approximately 25% of methane emissions from all sources (Saunois et al. 2024), and moreover peatlands are hotspots for methane oxidation by predominately aerobic methane‐oxidising bacteria (Chowdhury and Dick 2013; Dedysh 2009; Fechner and Hemond 1992; Nedwell and Watson 1995; Krumholz et al. 1995). More recently, these ecosystems have also been recognised as important sources of myriad biogenic volatile organic compounds (BVOCs) such as terpenoids and oxygenated compounds, of which isoprene is the major component (Rinnan et al. 2014; Seco et al. 2022). These ecosystems, in particular, are likely to be profoundly affected by human activity, in terms of changing climate and land use, which modify temperature, hydrology and nutrient availability, among others, in turn affecting biological diversity and productivity (Box et al. 2019; Allen et al. 2024). These changes influence the fluxes of greenhouse gases such as carbon dioxide, methane and nitrous oxide, but also of BVOCs, which have an indirect but nevertheless important effect on climate (Peñuelas and Staudt 2010; Laothawornkitkul et al. 2009; Yuan et al. 2024; Laine et al. 2019; Wang et al. 2024; Valolahti et al. 2015; Kramshøj et al. 2016; Vettikkat et al. 2023; Rinnan et al. 2020), by reacting with hydroxyl and nitrate radicals and ozone, both extending the lifetime of methane and also resulting in formation of secondary organic aerosols and cloud condensation nuclei (Boy et al. 2022; Shrivastava et al. 2017).

Isoprene (2‐methyl butadiene) is produced globally at the highest rate of all BVOCs and is released to the atmosphere at a rate approximately equal to emissions of methane (Guenther et al. 2012), although its rapid photooxidation results in atmospheric concentrations three orders of magnitude lower (Greenberg et al. 1999; Wiedinmyer et al. 2005). Isoprene is produced by some bacteria, by most animals, and by industrial processes (Murrell et al. 2020), but its primary source is plants, of which all major groups contain isoprene emitters, including wetland‐associated plants such as mosses and sedges (Sharkey et al. 2013; Hanson et al. 1999; Ekberg et al. 2011). Isoprene production protects plants from thermal damage and ozone and other oxidative stresses and is influenced by factors such as CO_2_ concentration and water availability, so emissions are dependent on environmental conditions (Guenther et al. 1993; Monson et al. 2012; Sharkey et al. 2008).

As well as a great deal of research into isoprene emission by plants, several studies have identified microbial isoprene uptake close to the sources of emission, in soils and in association with isoprene‐emitting plants, and many bacteria with this capability have been isolated and characterised (Murrell et al. 2020; Cleveland and Yavitt 1997, 1998; Gibson et al. 2021; Uttarotai et al. 2022; Singh et al. 2021; Carrión et al. 2020a; Dawson et al. 2021), although in this respect peatland ecosystems are underexplored. The only isoprene‐degrading pathway so far described in detail relies on a multi‐component iron‐containing isoprene monooxygenase (IsoMO) related to methane, toluene and alkene monooxygenases (van Hylckama Vlieg et al. 2000; Sims et al. 2022), whose product is isoprene epoxide. Two glutathione transferases (IsoI and IsoJ), a dehydrogenase (IsoH), a co‐enzyme A transferase (IsoG) and an aldehyde dehydrogenase (AldDH) are responsible for the subsequent catabolic steps and entry into central metabolism, reviewed by Dawson et al. (2023). The genes encoding IsoMO (*isoABCDEF*) and the enzymes performing the subsequent steps (*isoGHIJ*, *aldDH*) are invariably clustered together, enabling identification of isoprene‐degrading potential in bacterial genomes and metagenomes (Crombie et al. 2018).

While many studies have examined the isoprene emission rates of ecosystems or of individual plant species, and have often investigated the effects of changes in environmental factors such as water stress, temperature, light levels, or CO_2_ and ozone concentration, in general these report the net emission rates and do not attempt to separate the plant production from microbial consumption (Hakola et al. 1998; Niinemets et al. 2010; Potosnak et al. 2013; Yuan et al. 2016; Vedel‐Petersen et al. 2015; Brilli et al. 2007; Centritto et al. 2011; Geron et al. 2001; Janson and De Serves 1998). Clearly, the plants and the microbes inhabiting the phyllosphere may be differentially affected by both long term and transient environmental conditions. Thus, accurate prediction of BVOC emissions in response to changing conditions requires an understanding of the complex interplay between emission and consumption by the various ecosystem components (Bardgett et al. 2008; Cavicchioli et al. 2019; Zhou et al. 2012; Andersen et al. 2013; Jassey et al. 2013; Deslippe et al. 2012; Jansson and Hofmockel 2020).

Although IsoMO performs the essential first step in the pathway enabling microbial growth on isoprene, several enzymes can fortuitously co‐oxidise isoprene. For example, the soluble methane monooxygenase (sMMO) of many methane‐oxidising bacteria (methanotrophs) has an exceptionally wide substrate range and apart from methane is capable of oxidation of alkanes, alkenes and aromatics, none of which can support growth (Colby et al. 1977; Jiang et al. 2010). Both iron‐containing alkene monooxygenase and the copper‐containing monooxygenases, including particulate methane monooxygenase (pMMO) of methanotrophs and ammonia monooxygenase (AMO), also co‐oxidise a range of alkanes and alkenes (Hyman et al. 1988; Burrows et al. 1984; Fosdike et al. 2005) and indeed microbes with enzymes from all these groups are capable of isoprene oxidation (Dawson et al. 2020; Sims et al. 2023). Thus, co‐oxidation of isoprene by methanotrophs and nitrifiers might be a significant process in environments where they are abundant, such as peatlands. If this were the case, then climate‐induced changes in the abundance or activity of methanotrophs might have unexpected effects on isoprene emissions.

Alkynes are known inhibitors of monooxygenases, including sMMO and pMMO, AMO, alkene monooxygenase and toluene monooxygenase, which in some cases are differentially inhibited by alkynes of different chain lengths (Fosdike et al. 2005; Hyman and Daniel 1988; Yeager et al. 1999; Keener et al. 2001; Prior and Dalton 1985; Wright et al. 2020). Previous work with alkene monooxygenase and toluene monooxygenase, the enzymes most closely related to isoprene monooxygenase, showed that they were poorly inhibited by acetylene, (a potent inhibitor of sMMO and pMMO as well as bacterial and archaeal AMO), in comparison with longer chain‐length alkynes (Yeager et al. 1999; Wright et al. 2020; Ensign et al. 1992). In accordance with this, our previous work showed that IsoMO was inhibited by 1‐octyne, but not by acetylene, whereas neither pMMO nor sMMO was greatly inhibited by 1‐octyne, suggesting a method by which oxidation of isoprene by IsoMO and co‐oxidation by methanotrophs could be distinguished (Dawson et al. 2020; Sims et al. 2023; Wright et al. 2020).

The aims of this study were first to identify the isoprene‐oxidising potential of the microbial community associated with *Sphagnum* moss from an ombrotrophic bog, and to distinguish between isoprene production by the plant and the net isoprene release when this consumption was included. Second, our aim was to determine if isoprene uptake was due solely to microbes capable of utilising this volatile for both energy and carbon, or if co‐oxidation by other microbes, for example, by abundant methanotrophs, was an important contributor. Third, we aimed to identify the genetic potential for isoprene metabolism associated with the moss and to classify the isoprene degraders in the context of known isolates.

## 2 Experimental Procedures

### 2.1 Sampling

Material was obtained from Dersingham Bog, a Site of Special Scientific Interest near King's Lynn, Norfolk, UK, with the permission of Natural England. The bog area of the site is an acidic lowland mire, supporting a wide diversity of plants and animals. The bog is waterlogged during most of the year and is characterised by vegetation including bryophytes and tussocks of moorgrass ( *Molinia caerulea* ) and cotton grass ( *Eriophorum angustifolium* ). The mosses comprise mainly *Sphagnum* spp., but include *Polytrichum commune* , and are interspersed with small pools of water (Royles et al. 2022; Stevenson and Masson 2016). The *Sphagnum* species were identified previously as including *S. capillifolium* ssp. *rubellum*, *S. cuspidatum* , *S. fallax* and *S. papillosum* (Royles et al. 2022).

*Sphagnum* moss was sampled from a region, at approximately water‐table level, where a hummock rose from out of a pool. An area of approximately 100 × 200 mm was removed, including the growing moss, which was mostly above the water level, and approximately 75 mm depth of underlying peat from below the water level. The material was placed in polythene bags together with some pool water and air headspace, sealed and transported to the laboratory immediately. Pool water was also sampled and sealed in sterile polypropylene bottles (1 L volume) approximately half full. Material, sampled from the boardwalk to avoid disturbing the environment more than necessary, was obtained from the same location (grid reference 52.49.47 N, 0.28.19 E) on four occasions for DNA sequencing and microcosm experiments. The pH of the bog water, measured in the laboratory, was in the range 3.5–4.2 for all samples.

Initially, we were unsure if mosses harboured isoprene‐degrading microbes, and therefore we undertook a preliminary investigation to establish if there was isoprene uptake and if the 16S rRNA and *isoA* gene sequences of known isoprene degraders were present (May 2021, *Amplicon Sequencing*). However, as we found that *isoA* sequences similar to extant isoprene degraders were rare, we obtained more material for long‐read metagenomic sequencing (April 2023, *Long‐Read Metagenomic Sequencing*). Having established that there was isoprene uptake, we obtained material to investigate uptake rates (May 2022, *First Incubations*). Then, since results indicated that we should carry out additional experiments, as described below, we sampled again (October 2023, *Second and Third Incubations*) to investigate the potential for uptake of methane and uptake and production of isoprene by living moss and underlying peat in more detail.

For DNA extraction, moss was either used immediately or frozen at −20°C. Moss samples for incubations were kept in sealed polythene bags (with air headspace) at room temperature and bog water was stored at 4°C. All experiments were conducted with green moss shoots which had been growing above the water level, with additional bog water (either as‐sampled or filter‐sterilised) and with or without peat, as described below.

### 2.2 Amplicon Sequencing

Since we anticipated that isoprene was mainly produced by photosynthetically active material, DNA was extracted from the green, living parts of the sampled *Sphagnum* moss and also from similar moss following incubation with isoprene, in order to enrich isoprene‐metabolising microbes. For enrichment, a few grams of moss shoots were placed in 120 mL serum vials with 5–10 mL of bog water. Isoprene was added to sealed vials to 20–30 ppmv and uptake followed using a Fast Isoprene Sensor, as described below. Having established the approximate rate of isoprene uptake and to supply sufficient isoprene while maintaining this relatively low concentration, the vials were opened and placed in a 25 L glass bottle and isoprene was again added to 20–30 ppmv. The bottle was sealed with a rubber stopper and incubated at room temperature in the laboratory for 33 days. Approximately 0.5 g (wet weight) of moss was used for DNA extraction from the original moss (as sampled) and following enrichment, using a FastDNA Spin kit for Soil (MP Biomedicals, Irvine, CA, USA) followed by purification using a OneStep PCR inhibitor removal kit (Zymo Research, Irvine, CA, USA). Primers 341F/785R were used to obtain 16S rRNA gene amplicons (Klindworth et al. 2013), while *isoA* amplicons used primers isoA14F/isoA511R with cycling conditions as previously described (Carrión et al. 2018). Duplicate reactions were combined and sequenced as previously described (Carrión et al. 2020b). Briefly, 16S rRNA gene libraries were prepared and sequenced at Molecular Research LP (Shallowater, TX, USA) using Illumina MiSeq technology and a proprietary analysis pipeline, obtaining 170,000–200,000 reads per sample (300 nt paired end) (Table S1). We could not obtain an *isoA* PCR product from the moss as sampled but *isoA* amplicons from enriched samples were sequenced, resulting in 101,000 reads (Table S1). Analysis followed the DADA2 pipeline (v1.6) (Callahan et al. 2016). Reads were demultiplexed, primer sequences removed and reads were trimmed to 275 nt. Reads were quality filtered, denoised, dereplicated and paired reads were merged. Chimeric sequences were discarded and amplicon sequence variants (ASVs) were assigned. ASVs were checked against the NCBI nr database using BLASTx (Altschul et al. 1990) and those without homology to *isoA* were discarded, yielding 20 ASVs.

### 2.3 Long Read Metagenomic Sequencing

Since we suspected that novel *isoA* sequences might exist in the *Sphagnum* metagenome, DNA was again extracted from green parts of the moss (1.75–4.5 g wet weight). The moss was ground in liquid nitrogen and DNA was extracted using a method based on Porebski et al. (1997), except that chloroform/isoamyl alcohol was used instead of chloroform/octanol. The DNA, which was contaminated with humic acids and other inhibitory substances, was purified by caesium chloride ultracentrifugation. Three extractions were combined and the DNA (37 μg) resuspended in 4.0 g of Tris buffer (5 mM, pH 8.5). Caesium chloride (4.3 g) was added and gently dissolved, followed by 200 μL ethidium bromide (10 mg mL^−1^). Following centrifugation (275,000*g*, 20°C, 24 h, Vti 65.2 rotor (Beckman Coulter, UK), the visible band containing the DNA (approx. 1 mL) was withdrawn with a needle pierced through the side of the tube. Ethidium bromide was removed by multiple extractions with Tris‐saturated butanol and the DNA was precipitated with glycogen (1 μL) and two volumes of polyethylene glycol (PEG)/NaCl (30% PEG, 1.6 M NaCl) overnight. Following centrifugation (14,500*g*, 30 min, 22°C), the pellet was washed in 70% (v/v) ethanol, dried and re‐suspended in 100 μL Tris (5 mM, pH 8.5). Approximately 20 μg of high molecular mass DNA was submitted to Novogene (Cambridge, UK) for long‐read PacBio SMRT sequencing using a Sequel II sequencer. Due to the relatively low sequence coverage, attempts at sequence assembly were not successful. However, the resulting HiFi reads (Hon et al. 2020) were searched for genes of interest using tBlastn (Altschul et al. 1990) with amino acid sequences of IsoA (IsoMO alpha subunit), IsoI (glutathione‐*S*‐transferase), MmoX (soluble methane monooxygenase alpha subunit) and PmoA (particulate methane monooxygenase alpha subunit) as query sequences. Details of the sequence reads obtained are shown in Table S1.

### 2.4 Microcosms

Microcosms were set up to measure the rates of methane and isoprene uptake and the effects of the inhibitors acetylene and 1‐octyne. Methane (N5.5 purity) and acetylene were obtained from BOC (Woking, UK), isoprene (cat no. I19551) and 1‐octyne (244465) were from Merck (Gillingham, UK). All incubations were conducted in 120 mL glass serum vials at room temperature (20°C–22°C) exposed to daylight in the laboratory, except for incubations in a plant growth room as described below. The moss continued to grow in the vials and appeared healthy for the duration of all experiments. Killed controls were treated identically except that vials were autoclaved (121°C, 30 min) before the injection of substrate. Methane was added to 120 or 1000 ppmv (as indicated) and isoprene to 15–20 ppmv (headspace concentrations). These methane concentrations are at the lower end of those reported from similar environments which range from low micromolar to low millimolar in the aqueous phase (Zhang et al. 2021; Svensson and Rosswall 1984; Chasar et al. 2000; Shannon et al. 1996). The isoprene concentration was chosen with reference to measured or calculated concentrations (up to 30 ppmv) in the intercellular spaces of other plants for which data exist (Niinemets et al. 2010; Singsaas et al. 1997; Fall and Monson 1992; Brüggemann and Schnitzler 2002), since we anticipated that the microbes associated with isoprene uptake were internal to, or closely associated with the moss. Inhibitors acetylene or 1‐octyne were added to 50 μM liquid phase concentration, in accordance with our earlier work (Dawson et al. 2020; Sims et al. 2023; Wright et al. 2020). Liquid phase concentrations were calculated using the Henry's law constants of 1.3 × 10^−3^, 1.3 × 10^−2^, 4.2 × 10^−2^ or 1.2 × 10^−2^ M atm^−1^ for methane, isoprene, acetylene or 1‐octyne, respectively (Mackay and Shiu 1981; Hine and Mookerjee 1975).

### 2.5 First Incubations—Methane and Isoprene Uptake by Living Moss Shoots

Incubations were conducted with living (green) shoots of *Sphagnum* moss (approx. 2 g wet weight) plucked from the moss clumps and with any dead material removed, with 5 mL of bog water, in 120 mL serum vials. Vials (in triplicate for each condition) were sealed with butyl rubber stoppers and incubated with approximately 120 ppmv methane or 15–20 ppmv isoprene, added by injection through the septum as described previously (Crombie et al. 2015). To evaluate the effect of inhibitors on the uptake of isoprene or methane, additional incubations were included in which acetylene or 1‐octyne (in vapour form) was added after 5 h, by injection through the septum.

### 2.6 Second Incubations—Methane and Isoprene Uptake by Moss and Peat

**FIGURE 1:**
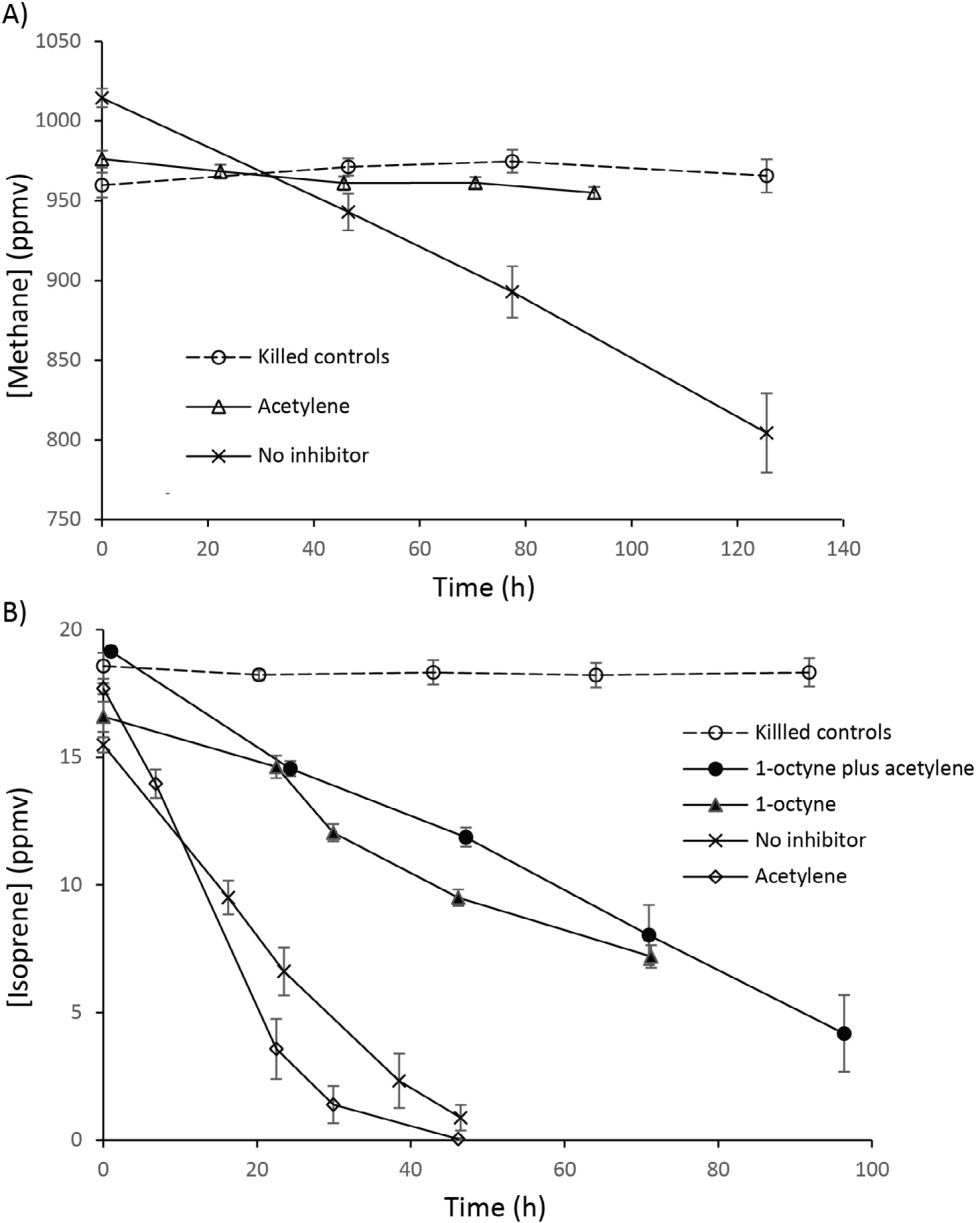
Consumption of (A) methane or (B) isoprene, by microcosms containing *Sphagnum* moss, peat and bog water, in the presence of the inhibitors acetylene or 1‐octyne, in comparison with no inhibitor (Second Incubations). Error bars show the SEM, *n* = 6 (live samples), *n* = 3 (killed controls).

Since we did not detect a rapid rate of methane uptake by *Sphagnum* microcosms in the first incubations, we repeated similar incubations in October 2023 but included the underlying decaying vegetation (peat) in case methane oxidation activity was localised to this material. Microcosms (15 identical vials with living material, plus three killed controls included to detect any abiotic loss, for example by leakage or diffusion through the stopper) contained 3.3 g peat, 10 mL of bog water and 4 g of green *Sphagnum* moss in 120 mL vials and were incubated with various additions sequentially. First, all vials were incubated with methane, at the increased concentration of 1000 ppmv. Methane uptake was monitored every 2–3 days in the killed controls and in six vials containing living material (plus three times during the incubation in the remaining nine vials, to verify that all vials containing living material consumed methane at similar rates). After 28 days, following consumption of methane in all vials, the vials were opened, flushed with sterile air, re‐sealed and isoprene was added (15–20 ppmv) to all vials. Vials containing isoprene used PTFE‐coated stoppers to reduce diffusive loss of isoprene through the rubber stopper. Microcosms consumed added isoprene over 48 h and the concentration was followed in the three killed control vials and in 6 experimental vials twice daily, and at the start and finish in the remainder, again to confirm a similar rate of isoprene uptake in all vials containing living material. Vials were again opened, flushed with air and isoprene again added. In addition, either acetylene or 1‐octyne (50 μM) was added (six replicate vials for each, plus three live and three killed controls which received isoprene only). Isoprene uptake was followed for 72 h. Finally, vials were again flushed, and vials which had contained acetylene were incubated with methane plus acetylene, whereas those which had contained 1‐octyne were incubated with isoprene plus 1‐octyne and acetylene, to further confirm that the addition of acetylene had no additional inhibitory effect. Live and killed controls received isoprene only as before. Isoprene uptake was followed in all 18 vials for 100 h. Since killed controls (three vials) were incubated with isoprene a total of three times, the data presented in Table 1 are the mean of all nine incubations for these controls, whereas for clarity Figure 1B shows the killed controls from three replicates.

**TABLE 1:** Methane and isoprene oxidation rates in vials containing *Sphagnum* moss and peat, with or without the inhibitors acetylene or 1‐octyne (second incubations).

Subs/inhib	None	Acetylene	1‐octyne	Acetylene plus 1‐octyne	Killed controls
Methane	0.548 ± 0.050 (*n* = 6) X, x	0.055 ± 0.009 (*n* = 6) Y, y	—	—	−0.018 ± 0.003 (*n* = 3) Z, z
Isoprene	0.100 ± 0.007 (*n* = 6) A, a	0.151 ± 0.006 (*n* = 6) A, d	0.037 ± 0.003 (*n* = 6) B, b	0.041 ± 0.005 (*n* = 6) B, b	0.008 ± 0.002 (*n* = 9) C, c

*Note:* The rates are shown as nmol of substrate h^−1^ (g of total material i.e., moss stems, water and peat)^−1^ ± SEM. Hyphens indicate that the combination was not tested. Different letters (X, Y, Z for methane, A, B, C, D for isoprene) indicate statistically significant differences in uptake rates between treatments (upper‐case, *p* < 0.001; lower‐case, *p* < 0.05).

### 2.7 Third Incubations—Isoprene Uptake and Production by Living Moss Shoots

To distinguish between plant isoprene production and microbial uptake, fresh microcosms were set up containing approximately 3–4 g green *Sphagnum* moss stems (exact mass recorded), without significant brown material or peat, in 120 mL vials with 10 mL of bog water sterilised by filtration (0.2 μM PES bottle‐top filter, Fisher Scientific, Loughborough, UK). Four conditions were set up, each in triplicate: no additions; with added isoprene (15–20 ppmv); with 1‐octyne (50 μM); with 1‐octyne (50 μM) but protected from light by wrapping the vials in aluminium foil. These vials were incubated in a plant growth room with a 12 h light/dark cycle (4,000 lx, 21°C).

### 2.8 Determination of Uptake Rates of Methane and Isoprene

Uptake of isoprene for amplicon sequencing was followed by injection of headspace gas into a Fast Isoprene Sensor (Hills Scientific, Boulder, CO, USA) as previously described (Sims et al. 2023). Due to the potential interference of acetylene or 1‐octyne with isoprene measurements, quantification of methane and isoprene in subsequent incubations was by gas chromatography (GC). Headspace gas (100 μL) was injected into an Agilent 7820A gas chromatograph fitted with a Porapak Q column (matrix 80/100, 6.0 ft. × 1/8 in × 2.1 mm (Supelco 14065‐U, Merck, Gillingham, UK) and flame ionisation detector. The injector was set at 200°C, oven 35°C or 175°C for methane or isoprene, respectively, detector 250°C. Standards were prepared by dilution of pure methane or by dilution of known concentrations of isoprene vapour (generated by addition of 2–50 μL of liquid isoprene into 1 L stoppered bottles using a glass syringe). The limit of accurate quantification for isoprene was approximately 0.25 ppmv. The identity of a compound generated by moss incubated without added isoprene but in the presence of 1‐octyne was confirmed by GC mass spectrometry (GCMS). Headspace gas was injected into a Shimadzu GCMS‐QP2010s gas chromatograph with quadrupole mass detector (Shimadzu, Milton Keynes, UK). The GCMS used a Porabond Q column (25 m × 0.25 mm × 3 μm, Merck, Gillingham, UK) with injection port at 200°C, oven 100°C for 3 min then ramped to 200°C at 8°C min^−1^ and held 4.5 min, interface 250°C, ion source 200°C, injection volume 50 μL. The machine was operated with a 5:1 split ratio in scanning mode (*m*/*z* 25–300), and isoprene eluted at 10.68 min. The spectra were compared with those of commercial isoprene (cat no. I19551, Merck Gillingham, UK) and the National Institute of Standards and Technology Mass Spectral Database NIST 08 library spectrum.

Uptake rates were derived from the slope of the linear least‐squares regression of methane or isoprene concentration over time. The regression for each experimental condition was fitted to all measured concentrations for the initial (linear) part of the uptake curve (15–48 data points per condition). Significant differences between treatments were assessed with two‐tailed *t* tests, applying the Bonferroni correction (Wright 1992).

The uptake rates are presented as either per gram of total material (peat plus water plus moss) or as per gram of living moss only, for comparison between incubations with or without peat, ± standard error of the mean (SEM).

## 3 Results

To investigate the potential of the microbial community associated with *Sphagnum* moss to consume added methane or isoprene, and the effects of possible inhibitors, we set up moss microcosms in glass vials and followed the consumption of these gases over time.

### 3.1 First Incubations—Live Moss Shoots Only

For these microcosms we used only the growing (green) moss stems without underlying peat. The moss consumed added isoprene (15–20 ppmv) rapidly and without an appreciable lag phase (Figure S1A), but added methane (120 ppmv) was not consumed rapidly under these conditions (Figure S1B). Incubations of *Sphagnum* with isoprene and the inhibitors of methane or isoprene oxidation, acetylene or 1‐octyne, either singly or in combination, showed that 1‐octyne but not acetylene inhibited isoprene oxidation under these conditions (Figure S1A). These experiments showed that isoprene was rapidly consumed by the *Sphagnum* microcosms, whereas these green *Sphagnum* moss shoots did not consume methane at a high rate.

### 3.2 Second Incubations—Live Moss Plus Peat

Since it seemed unlikely that these bog areas did not consume methane at appreciable rates, more material was sampled in the following year and microcosms were again set up as before with living *Sphagnum* moss, but also including the underlying peat and bog water (approximately 4 g, 3 g and 10 mL respectively) and supplied with methane (1000 ppmv). Vials consumed methane without an appreciable lag phase at a rate of 0.55 ± 0.05 nmol h^−1^ (g of total material)^−1^ (*n* = 6, ± SEM) (Table 1), suggesting that the active methanotrophs were residing in the peaty material rather than in the above‐water living shoots. Consumption of methane continued at an approximately linear rate until a concentration of approximately 200 ppmv was reached (corresponding to a liquid phase concentration of approximately 0.26 μM), and at a gradually decreasing rate thereafter (Figure S2). As expected, since acetylene is known to be a potent inhibitor of methane oxidation, inclusion of acetylene reduced the rate of methane oxidation by a factor of 10 (rate 0.055 ± 0.009 nmol h^−1^ (g of total material)^−1^ (*n* = 6, ± SEM)) (Figure 1A and Table 1). To assess isoprene uptake, methane‐consuming vials were opened, flushed with air, sealed and incubated with isoprene (15–20 ppmv, approximately 200 nM liquid phase concentration). Isoprene was consumed, also without an appreciable lag phase, at a rate of 0.092 ± 0.005 nmol h^−1^ (g of total material)^−1^ (*n* = 6, ± SEM) or, if only the mass of the green moss stems was considered, at a rate of 0.40 ± 0.02 nmol h^−1^ (g moss)^−1^, to below the limit of quantification (approximately 0.25 ppmv) within 2 days (Figure 1B and Table 1), similar to the rate of isoprene consumption in the first incubations comprising living moss shoots without peat (Figure S1A).

To evaluate the effect of inhibitors (acetylene or 1‐octyne) on isoprene oxidation, vials were opened, flushed with air, resealed and supplied with isoprene and acetylene, or isoprene and 1‐octyne. Vials containing acetylene again consumed isoprene at a rapid rate (0.15 ± 0.006 nmol h^−1^ (g of total material)^−1^ (*n* = 6, ± SEM)) (Figure 1B and Table 1), indicating that the methanotrophs and other microbes whose monooxygenases were inhibited by acetylene had little or no role in isoprene oxidation. The presence of 1‐octyne reduced the rate of isoprene oxidation by about 75%, but some residual isoprene uptake remained. When both 1‐octyne and acetylene were added to vials, isoprene was consumed at a similar rate to vials with 1‐octyne alone. Together, these data indicate that while the majority of isoprene oxidation was inhibited by 1‐octyne, a relatively minor amount was oxidised by non‐methanotrophic microbes, either *bona fide* isoprene degraders incompletely inhibited by 1‐octyne, or perhaps by others with enzymes capable of isoprene oxidation that were also not completely inhibited by either acetylene or 1‐octyne.

### 3.3 Third Incubations—Isoprene Uptake and Production by Living Moss Shoots

**FIGURE 2:**
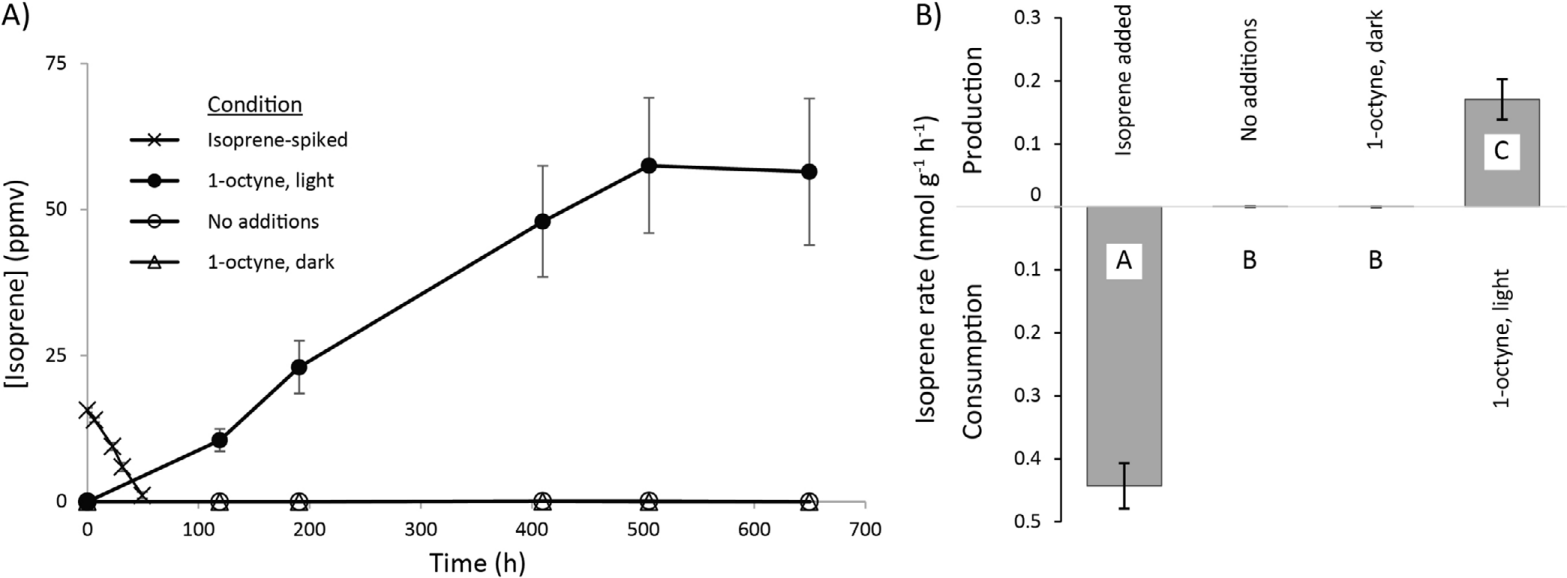
(A) Isoprene uptake or production by moss microcosms incubated under controlled lighting conditions, with or without the addition of isoprene, or without isoprene but with the inhibitor of microbial isoprene uptake, 1‐octyne, exposed to, or protected from light (Third Incubations). (B) the rates of isoprene production or consumption calculated from the data shown in panel A. Different upper‐case letters (A, B, C) indicate statistically significant differences between treatments (*p* < 0.001). The data show the mean ± SEM (*n* = 3).

To verify that the isoprene degraders resided in the growing (green) parts of the moss rather than in the peat below or in the bog water, green stems were plucked from the moss clumps and placed in vials without brown or peaty material, with filter‐sterilised bog water. Vials were incubated in a growth room under controlled lighting, and isoprene uptake was recorded (Figure 2A,B). Vials consumed isoprene at a rate of 0.44 ± 0.04 nmol h^−1^ (g moss)^−1^ (*n* = 3, ± SEM), almost identical to the rate reported above (0.40 ± 0.02 nmol h^−1^ (g moss)^−1^) when the latter is expressed as the rate per gram of green moss stems rather than the total mass (including water and peat) included in the former incubations, thus confirming that the living moss leaves and stems, rather than the underlying peat or the bog water, were mainly responsible for isoprene uptake.

To inhibit the microbes degrading isoprene on or in the moss, vials were incubated with 1‐octyne, but without added isoprene and exposed to light as described above. Under these conditions of restricted isoprene consumption by moss‐dwelling microbes, isoprene accumulated in vials at an approximately linear rate of 0.17 ± 0.03 nmol h^−1^ (g moss)^−1^ (*n* = 3, ± SEM) to a maximum of nearly 60 ppmv at 500 h, whereas control vials without the inhibitor only transiently accumulated isoprene to levels detectable by our system (Figure 2A,B). To verify that this was indeed isoprene which accumulated in 1‐octyne exposed microcosms, headspace gas was analysed by GC MS, revealing spectra identical to that of commercially obtained isoprene standards (Figure S3). To verify that this accumulation of isoprene was not due to any unknown effects of 1‐octyne on the moss plants, but rather was due to the lack of microbial isoprene uptake, control vials were incubated with 1‐octyne but protected from light, since plant isoprene production is dependent on light (Sharkey and Yeh 2001). Under these conditions, isoprene was mostly undetectable by our setup during the 650 h duration of the experiment (Figure 2A). The above value of 0.17 nmol h^−1^ g^−1^ therefore represents the minimum rate of isoprene production by the moss under conditions where microbial uptake was inhibited, and demonstrates that in un‐amended microcosms the microbes inhabiting the above‐ground parts of the moss consumed virtually all the moss‐produced isoprene, thus preventing accumulation in the vial headspaces.

### 3.4 Microbial Community Analysis

**FIGURE 3:**
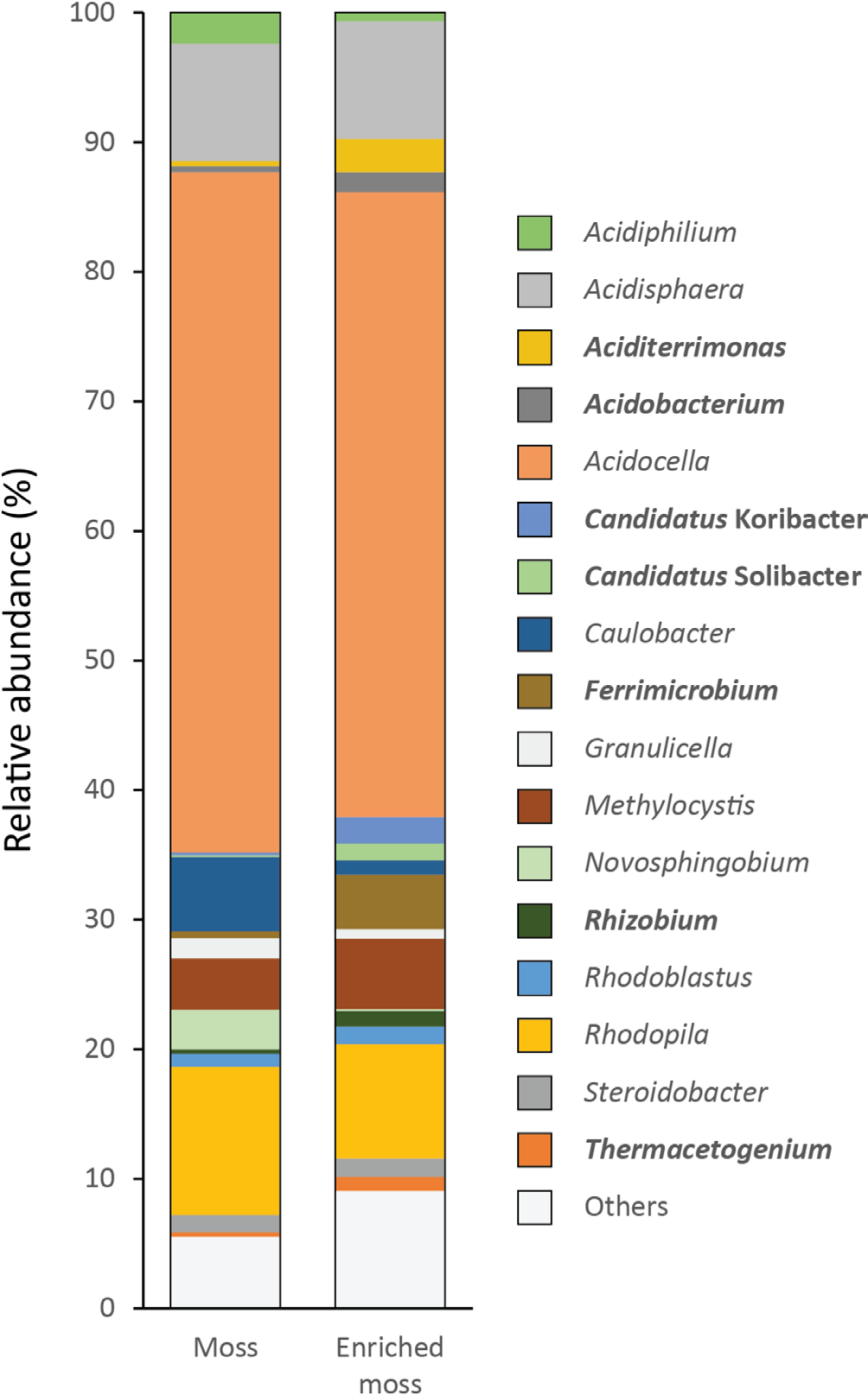
Microbial phylogeny based on 16S rRNA gene sequencing of *Sphagnum* moss shoots as sampled (Moss) and following enrichment with isoprene for 39 days (Enriched moss). Taxa increased in relative abundance over twofold between the two timepoints are shown in bold.

**FIGURE 4:**
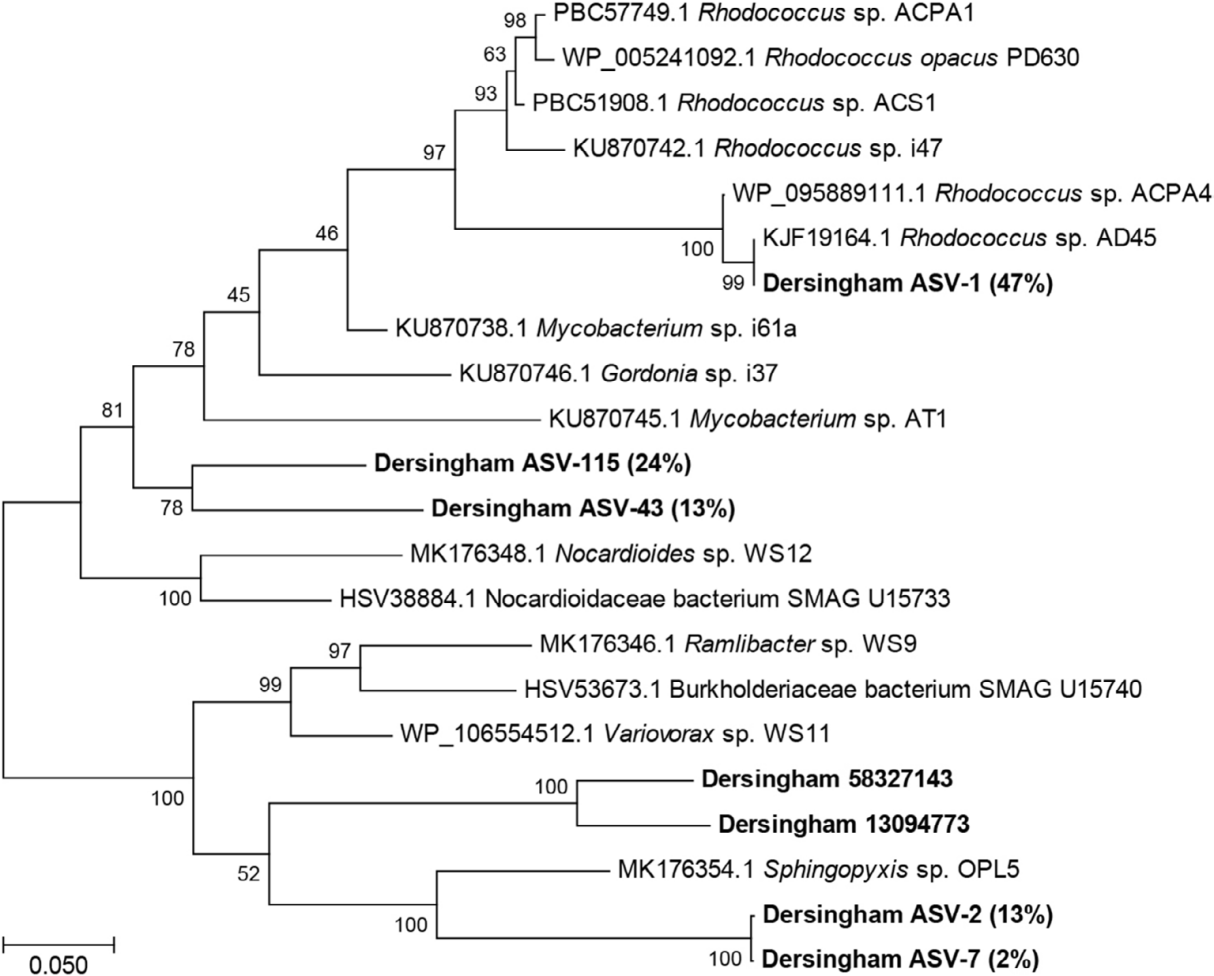
Phylogenetic tree showing the relationship between *isoA* sequences obtained from Dersingham Bog (in bold) with those of known isoprene degraders. The tree, constructed using the maximum likelihood method in Mega 7 (Kumar et al. 2016), is based on nucleotide sequences of the aligned proteins. Bootstrap values (500 replications) are shown at the nodes. The scale bar shows substitutions per site. The relative abundance of ASVs obtained in this study from amplicon sequencing of isoprene‐enriched samples is shown in parentheses, and two sequences from long read metagenomic sequencing are identified by their read numbers.

To characterise the *Sphagnum* moss microbial community, DNA was extracted and 16S rRNA gene amplicons were analysed by Illumina sequencing. The data (Figure 3) show that the moss community was dominated by alphaproteobacteria (93%), acidobacteriia (4%), gammaproteobacteria (2%) and acidimicrobiia (1%), typical of many *Sphagnum* communities (Andersen et al. 2013; Ivanova et al. 2020; Raghoebarsing et al. 2005). At the genus level, *Acidocella*, *Rhodopila*, *Acidisphaera*, *Caulobacter* and *Methylocystis* together comprised 83%, whereas representatives of genera previously associated with plant‐ or soil‐associated isoprene uptake such as *Rhodococcus, Mycobacterium, Variovorax, Ramlibacter* or *Sphingopyxis* (Murrell et al. 2020; Gibson et al. 2021; Crombie et al. 2018) were rare, together totalling approximately 0.1% of the bacterial community. As well as the unamended samples, DNA from moss enriched for 33 days with isoprene (15–25 ppmv) was also analysed. Various taxa, including *Ferrimicrobium*, *Candidatus* Solibacter and *Candidatus* Koribacter increased in relative abundance up to 10‐fold, but none of the groups that benefitted from the incubation were associated with previously identified isoprene degraders (Figure 3), except *Mycobacterium*, which increased sevenfold from an extremely low base (0.006%). To determine the diversity of isoprene degraders, DNA from the original and isoprene‐enriched samples was also challenged with primers designed to specifically target the *isoA* gene of all known isoprene degraders (Carrión et al. 2018). A PCR product of the expected size was not obtained from the unenriched samples, indicating that the abundance of microbes with DNA matching these primers was extremely low. The PCR reaction was successful, however, using DNA from enriched material, and yielded 20 ASVs with inferred amino acid identity of 87%–100% to the IsoA sequence of extant isoprene‐degrading isolates. Five ASVs, with abundance ≥ 1% of the total, together comprised 99% of the sequences, and their phylogenetic affiliations are shown in Figure 4.

### 3.5 Long‐Read Metagenomic Sequencing

**FIGURE 5:**
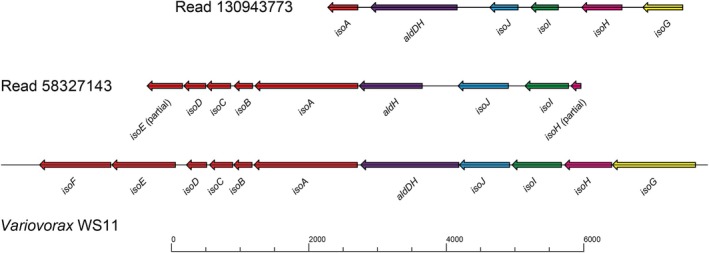
Gene sequences assumed to be responsible for isoprene degradation retrieved from DNA extracted from Dersingham moss. The *iso* gene clusters are aligned with those of reference strain *Variovorax* sp. WS11, a well‐characterised isoprene‐degrading isolate (Dawson et al. 2020; Larke‐Mejía et al. 2019).

Long‐read sequencing of DNA obtained by grinding the aerial parts of the moss in liquid nitrogen, to include both epiphytes and endophytes, identified two reads with both *isoA* and other genes associated with isoprene metabolism. Co‐localisation of the monooxygenase genes with genes encoding glutathione transferases required for isoprene metabolism is a feature in the genome of all known *isoA*‐containing isoprene degraders and thus provides compelling evidence of isoprene‐degrading potential (Murrell et al. 2020). Read 58327143 contained *isoHIJ‐aldH‐isoABCDE* and read 130943773 contained *isoGHIJ‐aldDH‐isoA* (Figure 5). The inferred IsoA sequences shared 77.5% and 80% amino acid identity with IsoA of *Ramlibacter* sp. WS9 (Larke‐Mejía et al. 2019) (96% identity to each other), and sequence alignment of the *isoA*, *isoI* and *isoJ* genes from Dersingham Bog revealed that they all grouped most closely with those of known Gram‐negative isoprene degraders such as *Sphingopyxis*, *Variovorax*, *Ramlibacter* and sequences in the databases including a Burkholderiaceae metagenome‐assembled genome (MAG) obtained via stable isotope probing of isoprene‐enriched soil samples (Larke‐Mejía et al. 2019; Ma et al. 2023), (Figures 4 and S4). Significantly, the primers used above for *isoA* amplicon sequencing contain mismatches to these sequences, including four or five mismatches (respectively) out of the six bases at the critical 3′ end of the reverse primer, meaning that these primers would probably not have amplified *isoA* from these microbes. Together these data strongly suggest that these are *bona fide* isoprene‐degrading gene clusters, although as the sequences are not closely related to those of isolated representatives, it appears that these environments harbour novel as‐yet unidentified groups of microbes with this metabolic trait.

## 4 Discussion

This study builds on previous work showing that isoprene‐degrading bacteria are inhibited by 1‐octyne but not by acetylene, in contrast with methane oxidizers (Dawson et al. 2020; Sims et al. 2023) and applies this principle to the moss‐associated microbial community in microcosms. Interestingly, despite *Sphagnum* being known to harbour abundant methanotrophs (Raghoebarsing et al. 2005; Kox et al. 2018), and detecting sequences affiliated to *Methylocystis* in our samples, our *Sphagnum* moss samples did not rapidly oxidise methane unless some of the peat soil and sediment were included in microcosms. Previously it was shown that submerged mosses were much more active in methane oxidation than above‐water material (Raghoebarsing et al. 2005; Putkinen et al. 2012; van Winden et al. 2012) and the data suggest that our non‐submerged living material did not contain abundant active methanotrophs or at least those with high methane‐oxidation potential. In contrast, when peat soil was included in microcosms, methane was indeed depleted at a significant rate, showing that methane oxidizers were abundant. The rate of methane uptake was reduced by 90% by acetylene, whereas isoprene uptake was not affected (Table 1), demonstrating that methanotrophs, at least in this environment, had little or no role in isoprene oxidation. In contrast, 1‐octyne (and a combination of 1‐octyne and acetylene) diminished isoprene uptake by 75%, similar to the degree of inhibition observed for the isoprene degrader *Rhodococcus* sp. AD45 (Sims et al. 2023) but considerably less than the 95% of inhibition observed in the case of *Variovorax* sp. WS11 (Dawson et al. 2020). While we do not discount the possibility that the remaining activity in the microcosms in the presence of 1‐octyne was due to only partial inhibition of *bona fide* isoprene degraders, it is also possible that a proportion of the isoprene uptake may have been accomplished by microbes not inhibited by either of these alkynes. For example, propylene monooxygenase of *Xanthobacter*, effectively inhibited by propyne, is capable of isoprene co‐oxidation, albeit with comparatively low affinity (Sims et al. 2023; Ensign 1996), but the effect of 1‐octyne is not known. It is possible that alkene monooxygenase‐containing microbes such as *Xanthobacter* were responsible for the residual isoprene uptake in the acetylene and 1‐octyne‐inhibited moss microcosms, or alternatively that the moss harboured microbes with alternative isoprene degradation pathways that are not inhibited by these alkynes (Gibson et al. 2021; Uttarotai et al. 2022).

Isoprene‐emitting plants have been said to typically divert 2% of photosynthesis to isoprene production (Sharkey and Yeh 2001), although the figure for *Sphagnum* mosses is not known. Here, independent sets of moss microcosms consumed isoprene at similar rates (Figures S1 and 2B and Table 1) suggesting that the isoprene‐oxidising potential of this moss is robust and consistent. Furthermore, inclusion of peat made no difference to isoprene oxidation rates (Table 1 and Figure 2B), suggesting that the isoprene consumers inhabited the living moss rather than the peat. The data presented in Figure 2 show that in these microcosms the microbes on or in the moss leaves consumed the vast majority of the moss‐produced isoprene, amounting in these microcosms to 0.17 nmol h^−1^ g^−1^ (wet weight). While we must, of course, exercise extreme caution in extrapolating these results more widely to other plants or ecosystems, the realisation that plant net‐isoprene‐emissions are, in some situations, overwhelmingly mediated by microbial isoprene degradation is highly significant. While in many ways reliant on each other, plants and microbes have different susceptibilities to environmental stresses and respond differently to changing conditions, both in the long and short term (Leveau 2019; Zhu et al. 2022), potentially altering the balance between isoprene production and uptake. It will be interesting to apply this approach to other isoprene‐producing plants, for example by including inhibitors in leaf‐chamber experiments to measure isoprene emissions from trees, thus quantifying the effect of phyllosphere microbes on isoprene emissions more generally. Given the major investment in isoprene production by many plants, the realisation that in some situations microbes may recycle this isoprene carbon, preventing its loss to the atmosphere, is significant for ecosystem carbon cycling.

Microbially mediated isoprene uptake in the plant phyllosphere has frequently been demonstrated in the past, and numerous isoprene‐degrading bacteria have been obtained from these environments (Gibson et al. 2021; Crombie et al. 2018, 2017; Carrión et al. 2020b; Larke‐Mejía et al. 2020; Singh et al. 2019). However, enrichments and stable‐isotope experiments have typically, with few exceptions, necessarily used elevated concentrations of isoprene, often orders of magnitude higher than those measured in the atmosphere or in the forest canopy, leading to uncertainty as to the ability of these microbes to consume isoprene at environmentally relevant concentrations. However, isoprene concentrations in or closely associated with leaves may be considerably higher (Niinemets et al. 2010; Singsaas et al. 1997; Fall and Monson 1992; Brüggemann and Schnitzler 2002), and the data presented in Figure 3, showing the accumulation of isoprene when microbial uptake was inhibited, prove that the microbes associated with the moss were able to consume isoprene at plant‐relevant concentrations.

Molecular analysis of DNA extracted from the moss identified a typical *Sphagnum*‐associated bacterial community, with few taxa associated with known isoprene‐degrading strains. The enrichment of some strains, notably *Ferrimicrobium*, *Ca*. Solibacter and *Ca*. Koribacter, by incubation with isoprene is interesting but inconclusive. Isoprene degraders from acidic or moss‐dominated environments have not to our knowledge been previously investigated, and the data, including the relatively dissimilar sequences retrieved from *isoA* amplicons (Figure 4) suggest that these strains may be novel isoprene‐degrading taxa, or alternatively that they may have benefitted indirectly from the input of isoprene carbon, perhaps by assimilation of the products of isoprene co‐metabolism by other members of the community. However, long read sequencing retrieved gene sequences unmistakably characteristic of the isoprene degradation pathway (Figure 5), albeit not closely related to extant examples, again suggesting that novel isoprene degrading strains may be prevalent in these environments. The data suggest that these are likely more closely affiliated with Gram negative strains rather than with isoprene degraders from the Actinobacteria (Crombie et al. 2017; El Khawand et al. 2016; Johnston et al. 2017) (Figures 4 and S4). Unfortunately, the sequence coverage obtained here, in the presence of the comparatively large moss genomes (Shaw et al. 2016), was insufficient to assemble the reads into metagenome‐associated genomes (MAGs), and phylogenetic identification of the isoprene degraders was not possible. A more comprehensive analysis, perhaps using targeted metagenomics following stable isotope probing as in our previous work (Crombie et al. 2018), should be a priority to identify the active isoprene degraders from wetland environments.

## 5 Conclusions

Here we show that *Sphagnum* moss, typical of environmentally sensitive wetland areas, harbours abundant specialist isoprene‐consuming microbes, and we show that the isoprene concentrations produced by the moss are sufficient for rapid microbial uptake. We demonstrate the use of specific inhibitors to differentiate between plant isoprene production and microbial consumption, quantify plant isoprene production and differentiate it from net emissions. We highlight that microbes may, in some circumstances, consume the overwhelming majority of plant‐emitted isoprene, preventing its escape to the atmosphere, which is important for predicting isoprene emissions in changing ecosystems. Furthermore, the data suggest that the isoprene degraders from this acidic environment belong to groups dissimilar to those previously known. The study introduces novel methods which can now be applied more widely.

## Author Contributions

**Andrew T. Crombie:** conceptualization (equal), investigation (equal), writing – original draft (lead), writing – review and editing (equal). **Chloe L. Wright:** conceptualization (equal), investigation (equal), writing – review and editing (equal). **Ornella Carrión:** investigation (equal), writing – review and editing (equal). **Laura E. Lehtovirta‐Morley:** funding acquisition (supporting), writing – review and editing (equal). **J. Colin Murrell:** conceptualization (lead), supervision (lead), funding acquisition (lead), writing – review and editing (equal).

## Conflicts of Interest

The authors declare no conflicts of interest.

## Supporting information


**Data S1.** Supporting Information.

## Data Availability

Sequence data are available at NCBI under accession number PRJNA272922: SRX26443778–80 (amplicons) and SRX26443781 (long reads).
