# Supplementary material for: Isoprene Production by Sphagnum Moss Is Balanced by Microbial Uptake, as Revealed by Selective Inhibitors
Source: Environ Microbiol. 2025 Jun 5;27(6):e70114. doi: 10.1111/1462-2920.70114 (PMC12141778; doi:10.1111/1462-2920.70114)
Supplement: Supplementary file 1 — Data S1. Supporting Information. [file EMI-27-e70114-s001.docx]

**Supplementary Appendix**

Supplementary Table S1. Sequence reads obtained in this study

| Description | Name | # Reads | Read length (nt) |  |
| --- | --- | --- | --- | --- |
| Amplicons |  |  |  |  |
| Unenriched 16S | M1.T0.16S | 198,474 | 300 |  |
| Enriched 16S | M1.E.16S | 171,630 | 300 |  |
| Enriched *isoA* | M1.E.isoA | 101,458 | 300 |  |
|  |  |  |  |  |
| Shotgun metagenomics |  | # Reads | Ave. length (nt) | N50 (nt) |
| PacBio HiFi reads | DM_hifi_01 | 38,683 | 2,925 | 3,640 |

Supplementary Figures


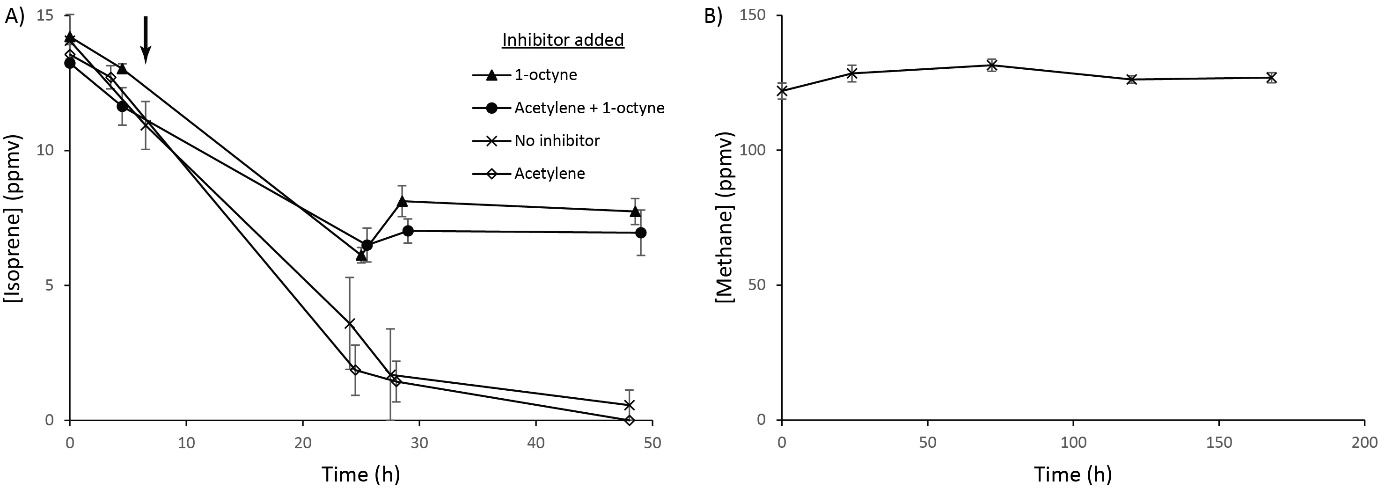


Supplementary Figure S1. First Incubations: A) Isoprene uptake by *Sphagnum* moss shoots either with no inhibitor, or in the presence of the inhibitors shown. The arrow shows the timepoint at which Inhibitors were added. B) *Sphagnum* moss shoots incubated in the presence of methane. Error bars show the SEM, n=3.


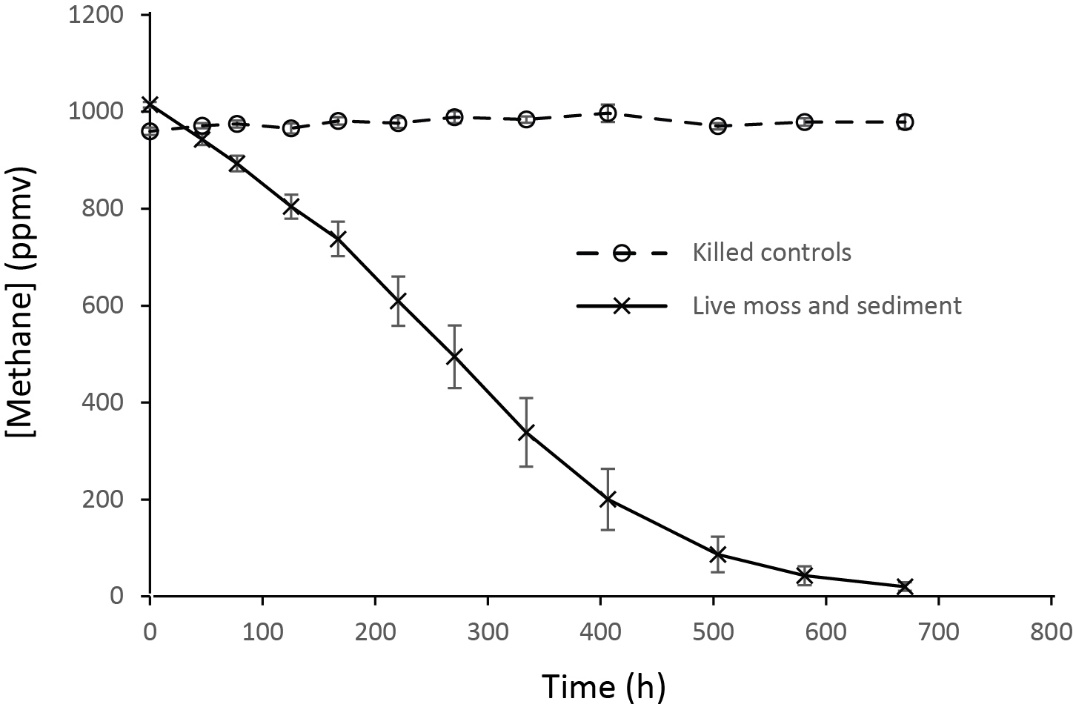


Supplementary Figure S2. Consumption of methane by microcosms containing live *Sphagnum* moss, underlying peaty material and bog water (second incubations). Error bars show the SEM, n = 6 (live samples), n = 3 (killed controls).


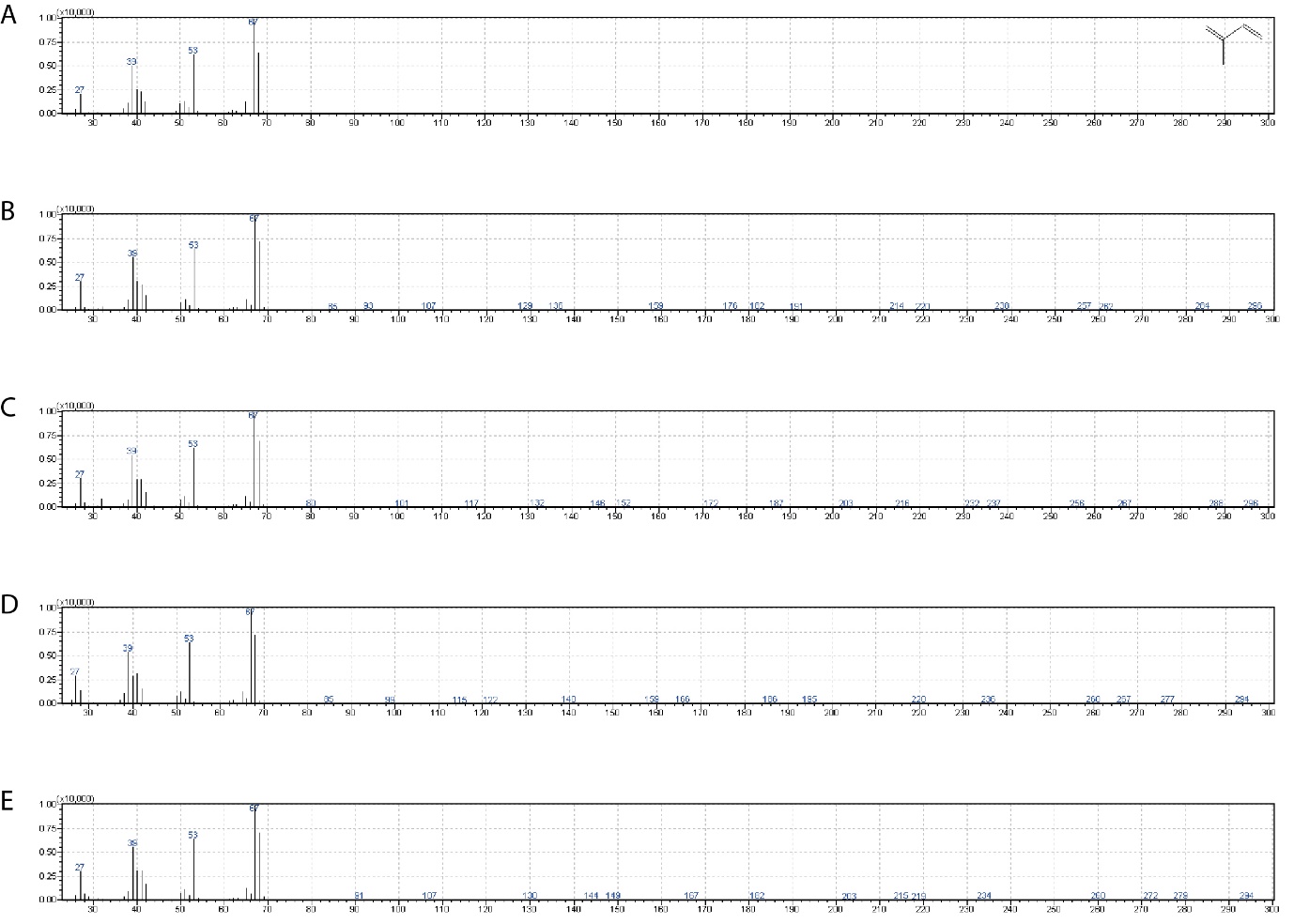


Supplementary Figure S3. Isoprene mass spectra. A), library spectrum; B), commercial isoprene (Sigma-Aldrich cat. no. I19551); C-E), replicate microcosm vials.


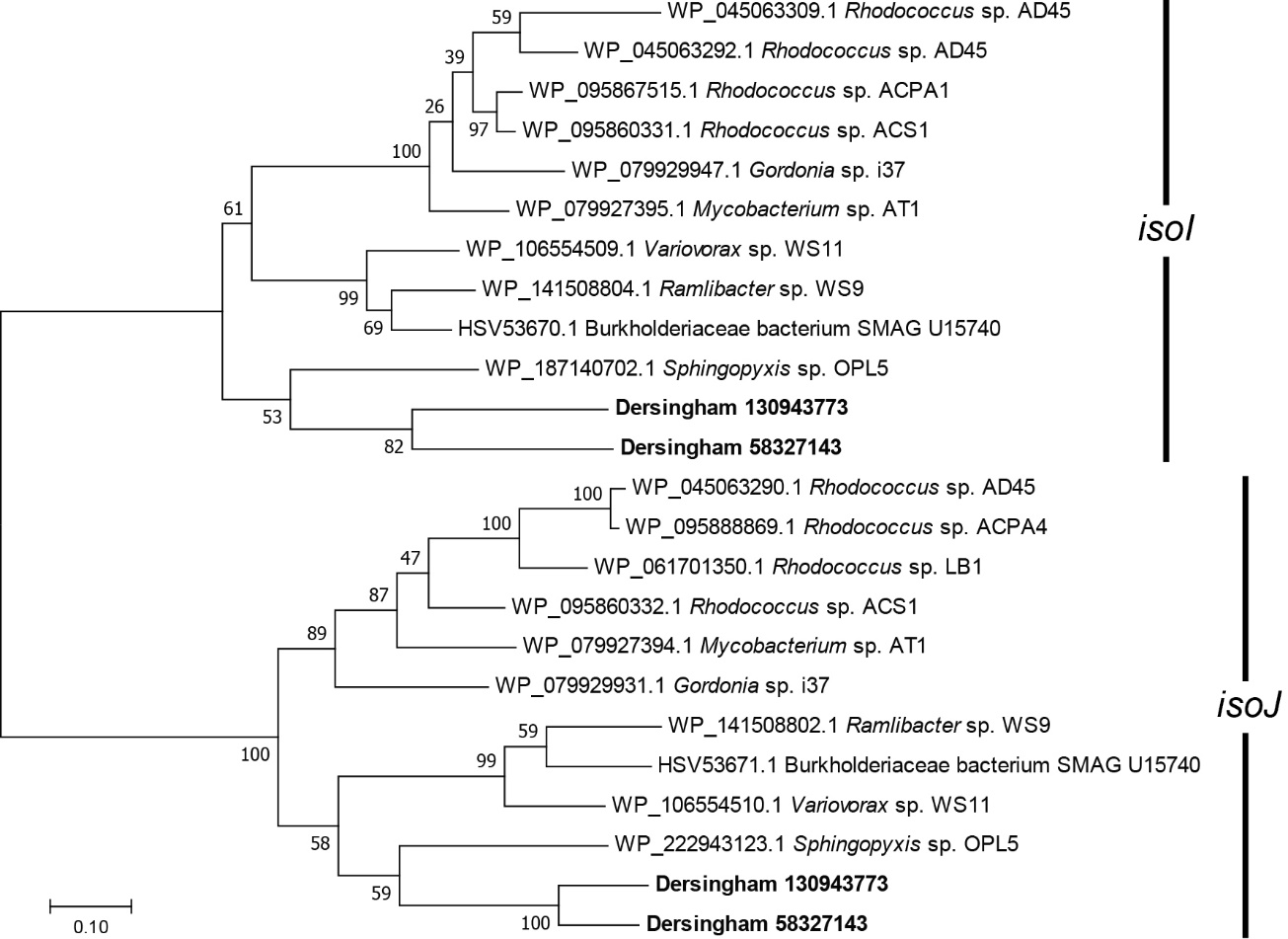


Supplementary Figure S4. Phylogenetic tree showing the relationship between *isoI* and *isoJ* sequences obtained from long-read metagenomic sequencing of DNA from Dersingham bog (in bold) with those of known isoprene degraders. The tree, constructed using the maximum likelihood method in Mega 7 (1), is based on nucleotide sequences of the aligned proteins. Bootstrap values (500 replications) are shown at the nodes. The scale bar shows substitutions per site.

1. S. Kumar, G. Stecher, K. Tamura, MEGA7: Molecular Evolutionary Genetics Analysis version 7.0 for bigger datasets. *Mol Biol Evol* **33**, 1870-1874 (2016).
